# Successful ECMO-cardiopulmonary resuscitation with the associated post-arrest cardiac dysfunction as demonstrated by MRI

**DOI:** 10.1186/s40635-015-0061-2

**Published:** 2015-09-03

**Authors:** Harald Arne Bergan, Per Steinar Halvorsen, Helge Skulstad, Thor Edvardsen, Erik Fosse, Jan Frederik Bugge

**Affiliations:** Department of Research and Development, Division of Emergencies and Critical Care, Oslo University Hospital, Nydalen, Oslo N-0424 Norway; Institute of Clinical Medicine, Faculty of Medicine, University of Oslo, Oslo, Norway; The Intervention Centre, Rikshospitalet, Oslo University Hospital, Oslo, Norway; Department of Cardiology, Rikshospitalet, Oslo University Hospital, Oslo, Norway; Department of Anaesthesiology, Division of Emergencies and Critical Care, Oslo University Hospital, Oslo, Norway

**Keywords:** ECMO-cardiopulmonary resuscitation, ECMO, Post-arrest, Left ventricular function, Cardiac MRI, Haemodynamics

## Abstract

**Background:**

Veno-arterial extracorporeal membrane oxygenation (ECMO-CPR) is a life-saving rescue for selected patients when standard cardiopulmonary resuscitation fails. The use is increasing although the treatment modality is not fully established. Resuscitated patients typically develop a detrimental early post-arrest cardiac dysfunction that also deserves main emphasis. The present study investigates an ECMO-CPR strategy in pigs and assesses early post-arrest left ventricular function in detail. We hypothesised that a significant dysfunction could be demonstrated with this model using magnetic resonance imaging (MRI), not previously used early post-arrest.

**Methods:**

In eight anaesthetised pigs, a 15-min ventricular fibrillation was resuscitated by an ECMO-CPR strategy of 150-min veno-arterial ECMO aiming at high blood flow rate and pharmacologically sustained aortic blood pressure and pulse pressure of 50 and 15 mmHg, respectively. Pre-arrest cardiac MRI and haemodynamic measurements of left ventricular function were compared to measurements performed 300-min post-arrest.

**Results:**

All animals were successfully resuscitated, weaned from the ECMO circuit, and haemodynamically stabilised post-arrest. Cardiac output was maintained by an increased heart rate post-arrest, but left ventricular ejection fraction and stroke volume were decreased by approximately 50 %. Systolic circumferential strain and mitral annular plane systolic excursion as well as the left ventricular wall thickening were reduced by approximately 50–70 % post-arrest. The diastolic function variables measured were unchanged.

**Conclusions:**

The present animal study demonstrates a successful ECMO-CPR strategy resuscitating long-lasting cardiac arrest with adequate post-arrest haemodynamic stability. The associated severe systolic left ventricular dysfunction could be charted in detail by MRI, a valuable tool for future cardiac outcome assessments in resuscitation research.

**Trial registration:**

Institutional protocol number: FOTS 4611/13.

**Electronic supplementary material:**

The online version of this article (doi:10.1186/s40635-015-0061-2) contains supplementary material, which is available to authorized users.

## Background

Patients with cardiac arrest not responding to standard cardiopulmonary resuscitation (CPR) can be resuscitated by veno-arterial extracorporeal membrane oxygenation (ECMO-CPR). Advances in technology and equipment have made ECMO-CPR much more attractive during recent years, and its use is increasing. The enthusiastic quest for novel resuscitation techniques may be due to the fact that standard CPR frequently fails. Although ECMO-CPR is claimed to represent “a frontier of resuscitation” [1], it is not an established treatment modality. No controlled clinical ECMO-CPR studies exist, but observational studies have been published [2–7]. Many centres have reported positively on their clinical experiences as reviewed recently by Fagnoul et al. [8]. Studies on ECMO-CPR usually focus on survival until hospital discharge and on post-arrest neurological sequelae. End-points in experimental studies are frequently the regain of spontaneous circulation (ROSC) and post-arrest brain tissue damage. A clinical problem after ROSC, however, is the early post-arrest cardiac dysfunction with circulatory failure-increasing the risk of adverse neurological function and poor outcome in resuscitated patients [9]. Detailed knowledge of cardiac function post-arrest is therefore needed. Few data exist on cardiac dysfunction after ECMO-CPR. The purpose of the present study was twofold; (1) to investigate ECMO-CPR in an experimental pig model and (2) to assess the early post-arrest left ventricular (LV) function by cardiac magnetic resonance imaging (MRI). We hypothesised that a significant early post-arrest LV dysfunction could be demonstrated with this model using MRI. Cardiac MRI allows accurate measurements of motions, volumes and flow in the beating heart and is not previously used to describe early post-arrest cardiac dysfunction.

## Methods

### Overall design and details

The study was a controlled experimental animal study. A listing of experiment details is presented in Tables 1 and 2.

**Table 1:** Animals and anaesthesia

Pig (sus scrofa domestica)	
Strain	Crossbreeding
Numbers included	8
Gender	3 female 5 male
Weight	49.7 ± 2.3 kg
Ventilation	
Mode	Volume control
Tidal volume	10 ml/kg
PEEP	5 cm H_2_O
Respiratory rate	18/min
I:E ratio	1:2
FiO_2_	40 %
Premedication (i.m)	
Azaperone	4 ml 40mg/ml (3 mg/kg)
Ketamine	30 ml 50mg/ml (30 mg/kg)
Atropine	1 ml 1mg/ml (20 μg/kg)
Anaesthesia (i.v)	
Pentobarbital	4 mg/kg/h
Morphine	2 mg/kg/h
Midazolam	0.15 mg/kg/h
Rocuronium	3 mg/kg/h
Euthansia (i.v)	
Pentobarbital	20 ml 50 mg/ml (20 mg/kg)
Morphine	5 ml 10 mg/ml (1 mg/kg)
Potassium chloride	50 ml 1 mmol/ml (1 mmol/kg)

Animals and anaesthesia used in the experiments

Weight as mean ± standard deviation; *PEEP* positive end-expiratory pressure, *I:E ratio* the ratio of inspiratory time (I) to expiratory time (E), *FiO*_*2*_ fraction of inspired oxygen, *i.m*. intra muscular, *i.v*. intra venous

**Table 2:** Equipment

Accessories	Name	Company	Location	
Ventilator	Leon Plus	Heinen & Löwenstein	Bad Ems	Germany
Ventilator in MRI	Fabius MRI	Drägerwerk AG & Co	Lübeck	Germany
Left ventricle pressure transducer	MPR-500	Millar Instruments	Houston, TX	USA
Venous vascular introducer	AVA High-Flow M3L9FHS 9Fr	Edwards Lifesciences	Irvine, CA	USA
Pulmonary artery catheter	Swan-Ganz CCO	Edwards Lifesciences	Irvine, CA	USA
Arterial vascular introducer	Radifocusintroducer II 8Fr	Terumo Europe	Leuven	Belgium
Per venous pacing lead	Qstim 5Fr	VascoMed GmbH	Binzen	Germany
Pulmonary artery monitor	Vigilance II Monitor	Edwards Lifesciences	Irvine, CA	USA
Pressure transducer software	Mikrosound Presenter 18.11.2011	Vestfold university college	Tønsberg	Norway
Defibrillator	CodeMaster XL+	Hewlett Pachard	Lexington, KY	USA
Data analysis software	Graphpad prism 6.04	GraphPad Software	La Jolla, CA	USA
Statistical software	SPSS v.22	SPSS	Chicago, IL	USA
MRI	Name	Company	Location	
MRI Scanner w/software	Philips Achieva 3 Tesla	Philips Medical Systems	DA Best	Netherland
MRI tagging analysis software	Diagnosoft HARP 3.0	Diagnosoft	Durham, NC	USA
MRI cine/phase-contrast image analysis	Segment version 1.9 R2585	Medviso AB	Lund	Sweden
ECMO circuit	Name	Company	Location	
Cannulae	Arterial: DLP Femoral 14Fr Venous: DLP Jugular 21Fr	Medtronic Inc	Minneapolis, MN	USA
Centrifugal pump	Biopump + BPX-80	Medtronic Inc	Minneapolis, MN	USA
Oxygenator	Affinity NT	Medtronic Inc	Minneapolis, MN	USA
Console	Biomedicus 550 Bio-console	Medtronic Inc	Minneapolis, MN	USA
Flow transducer	BioProbe TX50	Medtronic Inc	Minneapolis, MN	USA
Temperature probe	Model TP Series 400 Thermistor Probe	Medtronic Inc	Minneapolis, MN	USA
Heat-exchanger	Stöckert Heater-Cooler System 3T	Sorin Group	Milano	Italy
Oxygen/air mixer	Sechrist Model 20090	Sechrist Industries	Anaheim, CA	USA

Equipment used in the experiments

### Animal welfare

The experimental protocol was approved by the Norwegian National Animal Research Authority. The animal experiments were performed in accordance with the European Convention for the Protection of Vertebrate Animals used for Experimental and Other Scientific Purposes (European Council, ETS No. 170). Personnel who handled the animals were certified with Federation of Laboratory Animal Science Associations category C. Detailed information according to the ARRIVE guidelines (Animals in Research: Reporting In Vivo Experiments) [10] is presented in Additional file 1: Table S1. A pig model was chosen because of the close similarities to human cardiac anatomy and physiology, also adequate for the use of standard equipment and clinical MRI sequences.

### Animals and anaesthesia

Eight pigs were included in the study; each pig was kept fasting overnight-with free access to water, pre-medicated in the pig box housing and weighted before a short transport to the MRI research operating theatre. A mixture of intravenous anaesthesia standardised by weight and suspended in Ringer’s acetate solution was infused at 10 ml/kg/h (Table 1). The anaesthesia left no reaction to intermittent sharp hoof pinching or surgery and was chosen also to provide haemodynamic stability and the safe use of neuromuscular blockade [11, 12].

### Preparation

Each pig received a tracheostomy and intravascular catheters by a midline neck incision. A micro-manometer pressure transducer was positioned in the LV via an 8 French vascular introducer in the right carotid artery (Table 2). Both internal jugular veins were cannulated with 9 French vascular introducers and used to place a pulmonary artery (PA) catheter and a temporary pacing lead to the right ventricle. The femoral artery and the right external jugular vein were cannulated with 14 French and 21 French ECMO cannulae, respectively, using a guiding obturator placed over a guide-wire during fluoroscopy. To prevent ECMO clotting, a bolus of intravenous heparin 2 mg/kg followed by infusion 0.5 mg/kg/h was given to obtain an activated clotting time ≥300 s. Mechanical ventilation was set to keep PaO_2_ within 20 ± 5 kPa and PaCO_2_ within 5.0 ± 0.5 kPa. Each pig had standard monitoring during the experiment-including electrocardiography (ECG), pulse oximetry, end-tidal expiratory CO_2_, hourly urine output and oesophageal temperature.

### Experimental protocol

#### Baseline assessments

After preparation and a following 30-min stabilisation period, baseline cardiac MRI and haemodynamic measurements of LV function were obtained. An arterial and a mixed venous blood sample were also analysed.

#### Establishment of ECMO

After baseline assessments, the pig was connected to a custom-made femoro-jugular veno-arterial ECMO circuit (Table 2). The ECMO consisted of an 86 ml centrifugal blood pump and a 270 ml micro-porous polypropylene hollow fibre oxygenator with a 2.5 m^2^ surface area. An oxygen/air mixer enabled an adjusted sweep gas oxygen fraction and sweep gas flow rate. The circuit was without surface coating and primed with Ringer’s acetate solution. The machinery was initially set to standby with the vascular lines clamped.

#### Cardiac arrest and resuscitation

Ventricular fibrillation (VF) was induced by an electrical stimulator connected to the right ventricular pacing lead. VF was confirmed by ECG shape and aortic blood pressure drop, and the ventilator was set to standby. After 15 min of untreated VF, ECMO-CPR was initiated with initial blood pump speed at the maximum 4540 rounds per minute. The sweep gas was set at the same flow rate as the ECMO blood flow rate, the fraction of oxygen to 100 % and the circuit temperature to 38.0 °C (normothermia in the pig). After 5 min of ECMO-CPR, fibrillation was terminated by external damped sinusoidal waveform 360 Joule monofasic shocks through standard anterior/anterior pads until sinus rhythm was obtained. The extracorporeal support continued at the same blood flow rate for a total of 150 min. Repeated blood gas analyses were performed, and the sweep gas flow rate and the oxygen/air mixture were adjusted according to the protocol. The lowest acceptable mean aortic pressure (MAP) and pulse pressure after defibrillation and regain of spontaneous cardiac beating (ROSB) was set by protocol consensus to 50 and 15 mmHg, respectively. Intravenous adrenaline (epinephrine) 10–25 μg boluses were given to ensure these criteria were met initially. If in need of continued inotropic support, infusion of the beta-adrenergic agonist dobutamine 5 μg/kg/min was started [13], and the infusion rate was adjusted if necessary. Each pig was weaned from ECMO during a 60 min period with successive 0.7 l/min reductions in blood flow rate every 10 min, to zero support. A 60-min stabilisation period followed before post-arrest assessments.

#### Post-arrest assessments

At 285 min post-arrest, cardiac function was re-assessed by the PA catheter and the LV pressure transducer. Arterial and mixed venous blood samples were analysed, and a second cardiac MRI was made. Finally, the pig was euthanized.

### Cardiac MRI measurements

Image acquisition, interpretation and post-processing were done according to the recommendations from the Society for Cardiovascular Magnetic Resonance [14]. Cardiac MRI images were analysed by the software Segment version 1.9 R2585 (http://segment.heiberg.se) [15] and by Diagnosoft HARP 3.0.

The LV was assessed by adjacent parallel short-axis slices covering the full volume of the LV and by long axis slices. Series of cinematographic images of the cardiac cycle were recorded by retrospective ECG-gating without breath-holds. The images were analysed to estimate end-diastolic volume (EDV), end-systolic volume (ESV), stroke volume (SV = EDV-ESV), ejection fraction (EF = SV/EDV) and cardiac output (CO = SV · heart rate (HR)). In addition, mid LV radial wall thickening and mitral annular plane systolic excursion (MAPSE) were measured. Wall thickening was averaged from measurements in the septum and the lateral wall, and MAPSE averaged from antero-septal-and postero-lateral measurements.

Slices in short axis were also magnetically gridded throughout the cardiac cycle (tagging) to assess peak global systolic LV circumferential (Lagrangian) strain by harmonic phase analysis [16]. Strain assessment provides information on the process of myocardial deformation; it can be extended to include fine details [17], and it also known to quantify myocardial function during echocardiography [18, 19].

Phase-contrast imaging with blood velocity encoded through-plane images of the mid-ascending aorta was also included to assess LV stroke volume (SV_phase_) and cardiac output (CO_phase_). It has been stated that this technique “should provide the most accurate measurement available of cardiac output” [20].

For further descriptions of cardiac MRI measurements the reader is kindly referred to Additional files 2 and 3 with the accompanying illustrations in Additional file 4, 5, 6, 7 and 8.

### Haemodynamic measurements

The PA thermodilution CO measurements (CO_PA_) were performed with injections of 10-ml ice-cold isotonic glucose, and the CO_PA_ was calculated as the average value of three consecutive measurements. Central venous pressure (CVP) was measured, and the SV (SV_PA_ = CO_PA_/HR) and the systemic vascular resistance (SVR_PA_ = (MAP-CVP)/CO_PA_∙80) were calculated.

The LV pressure transducer measurements were recorded on a separate PC monitoring system and analysed offline with custom-built software based on a visual programming language (Table 2) [21]. The maximum systolic LV pressure (LVP_max_) and the maximum positive and negative first time derivate of LV pressure (dP/dt_max_ and dP/dt_min_) were measured, in addition to the end-diastolic LV pressure (EDP) and the end-systolic LV pressure (ESP). Systolic duration was measured from ECG R-peak to dP/dt_min_ and diastolic duration from dP/dt_min_ to onset of systole. Isovolumetric contraction time (IVC) was measured from ECG R-peak to dP/dt_max_. The isovolumetric relaxation constant tau was calculated using Weiss method [22] in a 50-millisec time-frame starting at dP/dt_min_. LV afterload was estimated from arterial elastance (Ea = ESP/SV).

### Measurements of oxygen consumption and oxygen delivery

The arterial and venous oxygen content (C_a_O_2_ and C_v_O_2_) were calculated from the arterial and mixed venous blood samples, respectively. Oxygen delivery (D_O2_ = CO∙CaO_2_) and oxygen consumption (V_O2_ = CO∙arterio-venous oxygen difference (C_a_O_2_ -C_v_O_2_)) were calculated.

**Fig. 1:**
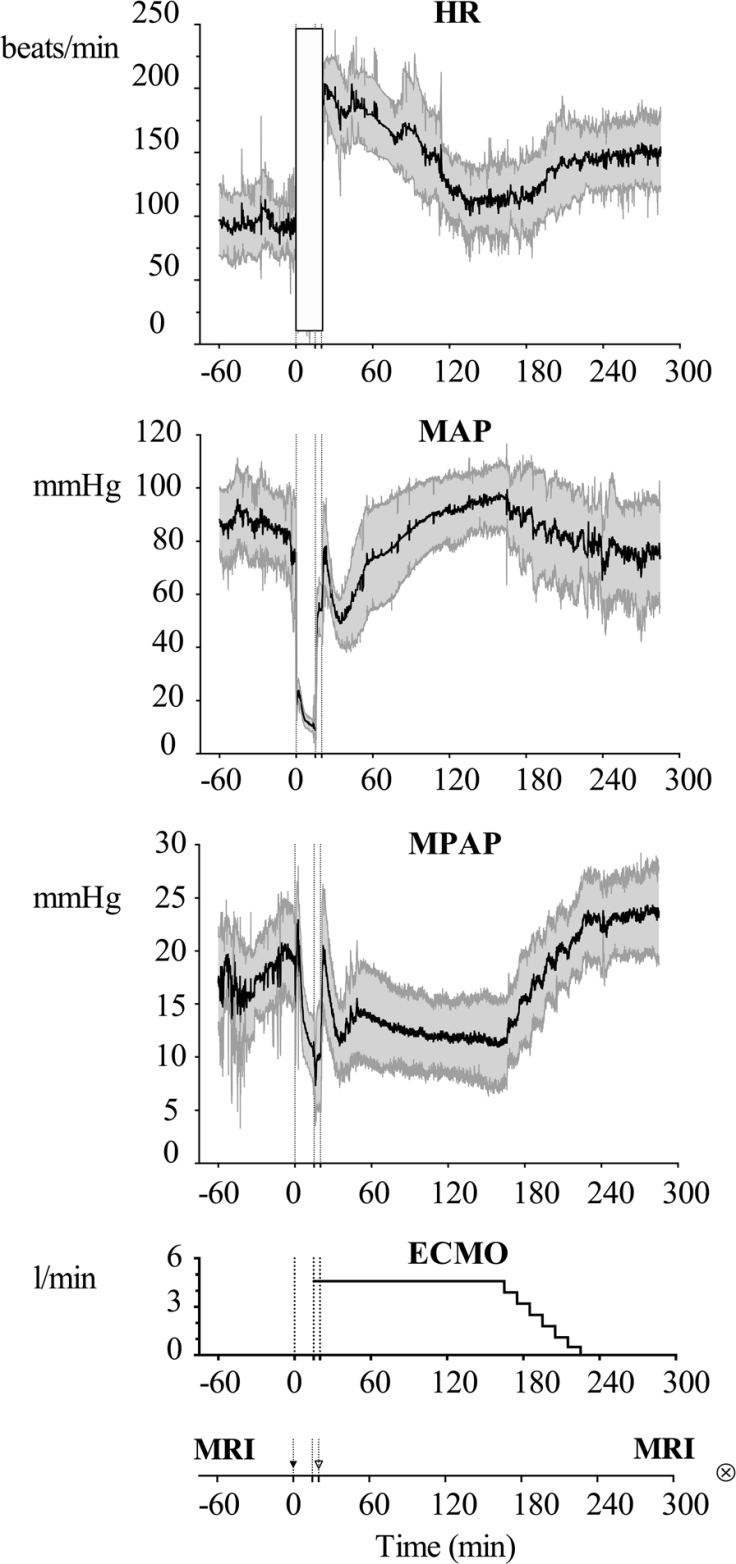
Continuous haemodynamic measurements (outside the MRI scanner). Continuous HR (heart rate), MAP (mean aortic pressure) and MPAP (mean pulmonary artery pressure) during the experiment, outside MRI scanner, shown as mean values (*black line*) ± standard deviation (*grey*). Cardiac arrest (VF) was induced at time 0 (*bottom timeline*, *black triangle* at *dotted line*). ECMO-CPR was started at time 15 (*separate dotted line*) and defibrillation was done at time 20 (*open triangle*). ECMO continued for 150 min before a 60-min stepwise weaning. Cardiac MRI of the beating heart was made ~60 min pre-arrest, and ~300 min post-arrest. The pig was euthanized after the post-arrest cardiac MRI (*bullet point*)

Outside the MRI scanner continuous measurements of HR, MAP and mean pulmonary artery pressure (MPAP) were recorded throughout the experiments (Fig. 1).

#### Statistical analysis

Statistical analyses of normality (D’Agostino-Pearson omnibus and Shapiro-Wilk) and of equality of variances (Levenes) supported the use of parametric tests. Data are reported as mean ± standard deviation if not otherwise stated. The statistical significance level *α* was set to 0.05. A paired two-tailed *t* test was used to compare baseline- and post-arrest measurements. The degree to which volumetrically and phase-contrast estimates of SV change correlated was quantified by a Pearson’s correlation analysis.

## Results

The pigs weighed 49.7 ± 2.3 kg, preparation took 119 ± 29 min and cardiac MRI scan time was 63 ± 15 min. Prior to induction of cardiac arrest activated clotting time was 349 ± 83 s, and ECMO circuit priming volume was 538 ± 17 ml. The experiments lasted 747 ± 71 min.

### Success of defibrillation

All animals were successfully defibrillated by a median of 1 shock (range 1–4). MAP at the time of defibrillation was 52 ± 8 mmHg with an ECMO blood flow rate of 4.60 ± 0.15 l/min. VF spontaneously reemerged after ROSB in two pigs demanding one additional defibrillation in both.

### Continuous haemodynamic measurements

Continuous HR, MAP and MPAP outside the MRI scanner are presented in Fig. 1. A considerable leap in HR was observed immediately following the defibrillation. The HR slowed gradually during the period of ECMO-CPR, but severe tachycardia with an approximate frequency of 150 beats/min remained throughout the experiment. MAP increased to a maximum of 77 ± 15 mmHg 2 min after defibrillation. MAP then dropped over the next 13 min to a low point of 49 ± 9 mmHg 20 min after initiation of ECMO-CPR. A further decrease in MAP was prevented by intravenous boluses of adrenaline and infusion of dobutamine. The median total dose of dobutamine and adrenaline was 10.3 mg (range 0–19.5 mg) and 80 μg (range 0–340 μg), respectively. MPAP changed in parallel with MAP to a maximum of 20 ± 5 mmHg 2 min after defibrillation. Both pressures stabilised as adrenaline and dobutamine were added. During the period of stepwise reductions in ECMO blood flow rate, MPAP increased in steps, reaching 22 ± 4 mmHg when weaning was completed. All animals could be successfully weaned from ECMO as dictated by the protocol. During the 60-min stabilisation period following ECMO separation, the haemodynamics remained unchanged (Fig. 1).

### Baseline and post-arrest haemodynamic measurements and blood gas values

Haemodynamic measurements and blood gas values at baseline and post-arrest are presented in Table 3. The substantial increase in HR post-arrest limited the reductions in CO. Systolic duration and isovolumetric contraction time were shortened by 33 ± 8 % and 17 ± 12 %, respectively, whereas diastolic duration decreased by 57 ± 17 %. LV afterload estimated by Ea increased post-arrest, and there was also a noticeable rise in MPAP. There was a minor increase in dP/dt_max_ whereas LVP_max_ did not significantly change; neither did EDP, dP/dt_min_ nor tau.

**Table 3:** Haemodynamic measurements and blood gas values

A. Pulmonary artery catheter measurements and MAP				
Variable	Baseline	Post-arrest	MD (95 % CI)	*p* value
HR_PA_ beats/min	86 ± 22	147 ± 28	61 ( 37 86)	*p* < 0.001
MAP mmHg	84.3 ± 13.1	74.6 ± 16.8	−9.6 (−27.0 7.8)	*p* = 0.23
CO_PA_ l/min	4.5 ± 0.6	3.8 ± 0.4	−0.72 (−1.5–0.08)	*p* = 0.07
SV_PA_ ml	53 ± 6	26 ± 3	−27 (−33–22)	*p* < 0.001
MPAP mmHg	17.8 ± 3.6	23.6 ± 4.3	5.9 ( 3.4 8.4)	*p* < 0.001
CVP mmHg	6.3 ± 2.6	6.3 ± 3.2	0.0 (−2.0 2.0)	*p* = 1.0
Wedge mmHg	8.7 ± 3.7	8.5 ± 3.4	−0.2 (−3.4 3.1)	*p* = 0.89
SVR dyne•s/cm^−5^	1390 ± 232	1438 ± 288	48 (−114 209)	*p* = 0.51
B. Left ventricle pressure transducer measurements				
Variable	Baseline	Post-arrest	MD (95 % CI)	*p* value
LVP_max_ mmHg	99.2 ± 7.1	91.4 ± 14.4	−7.9 (−23.4 7.6)	*p* = 0.27
EDP mmHg	15.5 ± 4.0	14.7 ± 2.5	−0.8 (−5.4 3.9)	*p* = 0.71
dP/dt _max_ mmHg/sec	1440 ± 209.5	2034 ± 549.4	594.1 (15.3 1173)	*p* = 0.046
dP/dt_min_ mmHg/sec	−2086 ± 285	−1715 ± 419	371 ( −185 926)	*p* = 0.16
tau msec	32.8 ± 3.0	33.5 ± 7.3	0.68 (−5.7 7.1)	*p* = 0.68
Ea mmHg/ml	1.04 ± 0.21	1.67 ± 0.47	0.63 (0.21 1.06)	*p* = 0.009
DD msec	440 ± 89	190 ± 40	−250 (−336–165)	*p* < 0.001
SD msec	328 ± 19	220 ± 19	−109 ( −133–84)	*p* < 0.001
IVC msec	50 ± 7	42 ± 6	−9 (−14–3)	*p* = 0.007
C. Blood gas analyses				
Variable	Baseline	Post-arrest	MD (95 % CI)	*p* value
Haemoglobin g/dl	8.4 ± 0.7	8.2 ± 0.8	−0.24 (−0.49 0.02)	*p* = 0.06
PaO_2_ kPa	22.6 ± 2.1	20.1 ± 3.5	−2.5 (−4.7–0.34)	*p* = 0.03
PaCO_2_ kPa	5.2 ± 0.4	5.3 ± 0.3	0.14 (−0.29 0.57)	*p* = 0.46
pH	7.50 ± 0.03	7.47 ± 0.04	−0.03 (−0.07 0.01)	*p* = 0.08
Base excess mmol/l	6.63 ± 1.25	4.73 ± 1.79	−1.9 (−3.02–0.78)	*p* = 0.005
Lactate mmol/l	0.88 ± 0.21	1.34 ± 0.54	0.46 (−0.03 0.95)	*p* = 0.06
Ca^2+^ mmol/l	1.29 ± 0.07	1.26 ± 0.07	−0.025 (−0.08 0.03)	*p* = 0.32
SvO_2_ %	69.4 ± 13.1	44.0 ± 7.9	−25.4 (−36.4–14.4)	*p* < 0.001
DO_2_ ml/min	572 ± 124	499 ± 119	−74 (−228 79)	*p* = 0.29
VO_2_ ml/min	164 ± 49	273 ± 46	109 (57 160)	*p* = 0.002

*HR*_*PA*_ *, CO*_*PA*_ *and SV*_*PA*_ pulmonary artery catheter assessments of heart rate, cardiac output and stroke volume, *MAP* mean aortic pressure, *CVP* central venous pressure, *Wedge* pulmonary arterial wedge pressure, *SVR* systemic vascular resistance, *LVP*_*max*_ systolic left ventricular pressure maximum, *EDP* end-diastolic pressure, *dP/dt*_*max*_ *and dP/dt*_*min*_ maximum and minimum LVP time derivates, *tau* isovolumetric relaxation constant, *Ea* arterial elastance, *DD* diastolic duration, *SD* systolic duration, *IVC* isovolumetric contraction time, *S*_*V*_ *O*_*2*_ mixed venous oxygen saturation, *D*_*O2*_ oxygen delivery, *V*_*O2*_ oxygen consumption

Values are expressed as mean ± standard deviation. *MD* mean difference, *CI* confidence interval, significance level *p* ≤ 0.05

Arterial blood gas values remained virtually unaltered post-arrest, and the haemoglobin concentration was stable (Table 3). The D_O2_ was maintained due to the increased HR, but mixed venous oxygen saturation dropped from 69.4 ± 13.1 % to 44.0 ± 7.9 % (*p* < 0.001) post-arrest, corresponding to a 66 ± 38 % increase in V_O2_ at almost unchanged D_O2_ (mean difference –74 ml/min (*p* = 0.29)).

### Baseline and post-arrest cardiac MRI

**Fig. 2:**
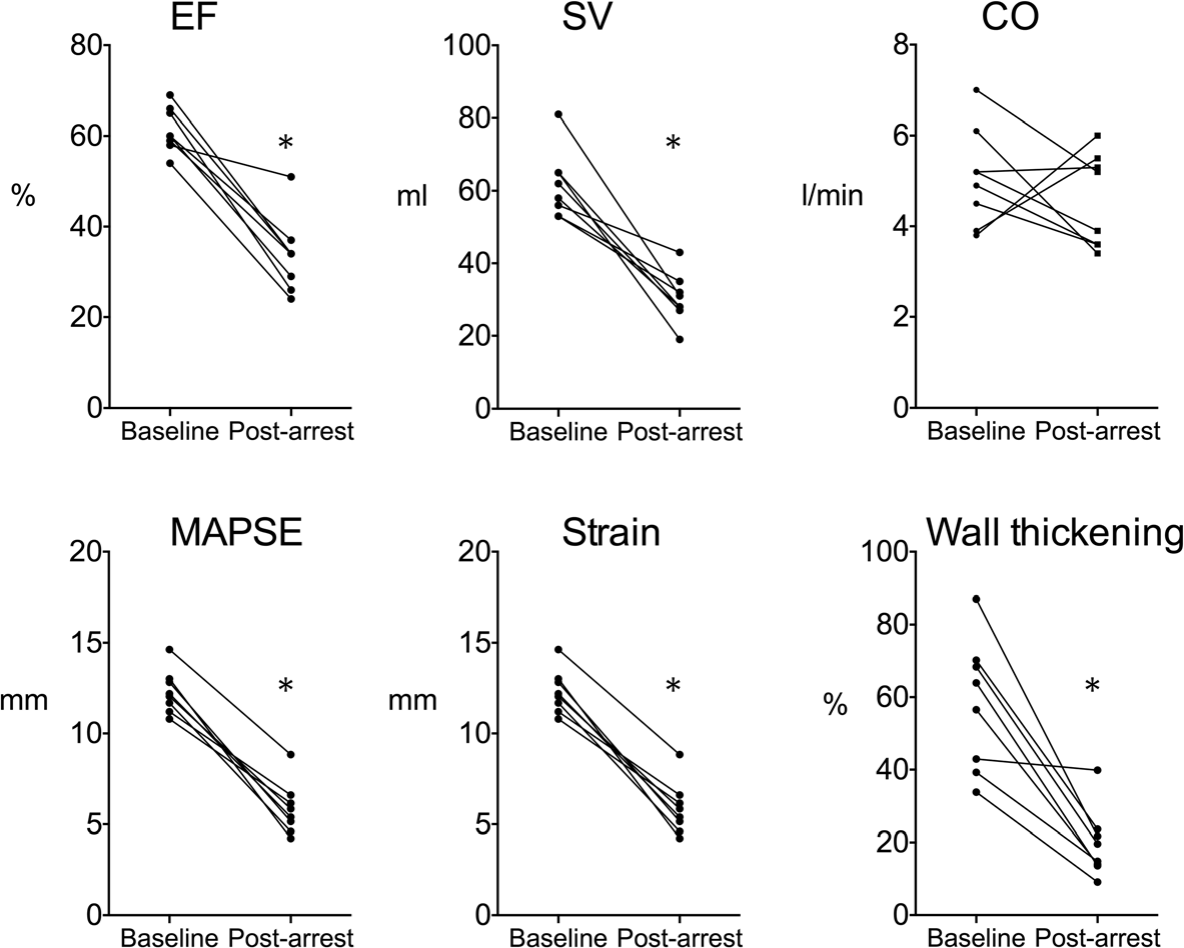
Baseline and post-arrest cardiac MRI. EF (ejection fraction), SV (stroke volume), CO (cardiac output), MAPSE (mitral annular plane systolic excursion), strain (peak global systolic left ventricular circumferential strain) and wall thickening before (*baseline*) and at ~300 min post-arrest. A paired two-tailed *t* test was used to compare baseline- and post-arrest measurements; asterisk: *p* value < 0.001

Cardiac MRI measurements at baseline and at 308 min ± 8 min post-arrest are presented in Table 4 and Fig. 2. LV function was severely reduced post-arrest, as EF decreased from 61 ± 5 % to 34 ± 8 % (*p* < 0.001) and SV decreased from 62 ± 9 ml to 30 ± 7 ml (*p* < 0.001). The lowered EF was related to a 58 ± 39 % increase in ESV as EDV was maintained (mean difference −8.9 ml (*p* = 0.22)). The reduction in SV was consistent with the change in SV_phase_ (Pearson *r* = 0.7). The minor reduction in CO (0.5–0.7 l/min) was not statistically significant. Consistent with the reduced SV the absolute value of global systolic circumferential strain decreased from −17.4 ± 4.2 % to −5.1 ± 3.3 % (*p* < 0.001) and MAPSE was reduced from 12 ± 1 mm to 6 ± 1 mm (*p* <0.001). Similarly, mid LV radial wall thickening was severely affected by a reduction from 58 ± 18 % to 20 ± 9 % (*p* < 0.001). The reductions in MAPSE and wall thickening were consistent between the different measuring locations in the LV.

**Table 4:** Cardiac MRI measurements

Variable	Baseline	Post-arrest	MD (95 % CI)	*p* value
HR_MRI_ beats/min	83 ± 19	153 ± 32	70 (36 103)	*p* = 0.002
CO_phase_ l/min	4.5 ± 0.6	3.8 ± 0.9	−0.7 (−1.7 0.3)	*p* = 0.12
SV_phase_ ml	56 ± 9	26 ± 9	−29 (−41–18)	*p* < 0.001
EDV ml	100 ± 10	92 ± 15	−8.9 (−25 7)	*p* = 0.22
ESV ml	39 ± 5	61 ± 15	23 (10 35)	*p* = 0.003
Wall thickening septum %	56 ± 18	17 ± 11	−39 (−53–25)	*p* < 0.001
Wall thickening lat %	59 ± 31	22 ± 10	−37 (−65–9)	*p* = 0.016
MAPSEantsept mm	10 ± 2	4 ± 1	−6 (−7 -4)	*p* < 0.001
MAPSEpostlat mm	15 ± 1	8 ± 2	−7 (−9 -5)	*p* < 0.001

*HR*_*MRI*_ heart rate during MRI, *CO*_*phase*_ *and SV*_*phase*_ cardiac output and stroke volume by cardiac MRI phase-contrast technique, *EDV* end-diastolic volume, *ESV* end-systolic volume, *Wall thickening*_*septum*_ *and Wall thickening*_*lat*_ mid left ventricular radial wall thickening in septum and lateral wall, *MAPSE*_*antsept*_ *and MAPSE*_*postlat*_ mitral annular plane systolic excursion measured anteroseptally and posterolaterally

Values are expressed as mean ± standard deviation. *MD* mean difference, *CI* confidence interval; significance level *p* ≤ 0.05

## Discussion

In this study, ECMO-CPR emerged as an effective tool resuscitating the heart from long-lasting cardiac arrest. Fifteen minutes of VF induced a severe early post-arrest LV dysfunction that was demonstrated in detail by cardiac MRI. This is the first study to describe early post-arrest LV dysfunction by cardiac MRI.

There are no ECMO-CPR guidelines for patients with refractory cardiac arrest. As a consequence, several strategies are implemented targeting various circuit flows, arterial pressures, oxygen saturations and core temperatures. Indications may also differ between centres, although a refractory normothermic arrest with a no-flow period of less than 5 min will generally be accepted for ECMO-CPR. A 60-min time-frame is often stated as the clinical accepted limit of low-flow (the period of standard CPR) before ECMO is established. In animals, without extracorporeal support the probability of successful ROSC decreases drastically when VF is untreated for more than 10–12 min, and it is almost impossible to achieve sustainable ROSC in pigs after 15 min of untreated VF [23–26]. In the present study, the 15 min duration of cardiac arrest followed by ECMO-CPR was chosen to allow early survival [23, 27–30], but with a significant post-arrest cardiac dysfunction that could be examined by cardiac MRI in a stable state.

### ECMO-CPR strategy

All pigs were successfully resuscitated by our protocol using a short (150 min) ECMO-CPR run with maximal flow, initially 100 % oxygen, normothermia, MAP above 50 mmHg and pulse pressure above 15 mmHg. The diameter, length and position of the venous cannula usually limit the ECMO blood flow rate. In our study, the venous cannula was placed as deep as the diaphragm, with its multiple side holes covering the full length of the caval veins and the right atrium. With this placement, the ECMO blood flow rate during ECMO-CPR could be kept as high as baseline CO pre-arrest without incidents of “suck down”, and increased CVP could be avoided. The low MPAP during ECMO-CPR indicated a relatively low blood flow through the pulmonary circulation, and thus a limited amount of blood entering the LV. This venous unloading, combined with adequate myocardial perfusion, is known to be particularly important to the post-arrest myocardium [31].

In peripheral veno-arterial ECMO, blood from the arterial cannula flows upwards in the descending aorta, increasing LV afterload. LV distension may then develop with compromised myocardial perfusion and hydrostatic pulmonary oedema. Although coronary perfusion was not measured, serious hypo-perfusion was most likely prevented by our protocol with the attention to adequate MAP and pulse pressure, the latter to assure LV emptying. The pressures were maintained above the lower limits by the customised dosage of positive inotropic medication. Dobutamine enhance LV function post-arrest [32]. All pigs could be weaned from extracorporeal support in accordance with the study protocol—this success would not have been possible after ECMO-CPR with severely compromised myocardial perfusion.

### LV function after ECMO-CPR

Cardiac MRI is often considered as the reference standard for the assessments of LV volumes and function. In addition, we assessed post-arrest LV haemodynamics by a PA catheter and a LV pressure transducer. Cardiac MRI demonstrated a severe impairment of LV function after ECMO-CPR. The systolic dysfunction was evident both by the global volume and flow measurements and by the LV motion measurements, mid LV radial wall thickening, MAPSE, and global systolic circumferential strain. Interestingly, post-arrest dysfunction was characterised by almost similar reductions (approximately 50–70 %) of systolic LV motion in all three deformation directions, indicating a global LV impairment.

When compared to cardiac MRI the LVP and dP/dt measurements did not reflect the severity of systolic LV dysfunction. LVP was maintained because MAP was pharmacologically sustained as prescribed by the protocol. An increased inotropic state post-arrest-a result of the dobutamine infusion and an endogenous catecholamine response [33, 34] probably contributed to the observed increase in dP/dt_max_ and HR [35, 36]. In addition, an increased afterload (Ea) post-arrest and a shorter systolic duration and IVC may also have influenced the dP/dt variable [37, 38]. The CO_PA_ and SV_PA_ measurements, however, corresponded well with the MRI measurements, but PA assessments gave less information.

In addition to the success of ECMO-CPR, our study demonstrates that a detailed assessment of early post-arrest LV dysfunction is feasible by cardiac MRI. The model may be relevant for future research on ECMO-CPR strategies and cardiac outcome.

Diastolic LV dysfunction was not demonstrated by the LV function variables included in this study. Post-arrest ESV increased without changes in EDV or EDP, indicating reduced systolic emptying of a ventricle adequately filled in diastole. A preserved diastolic function was supported by an unaltered tau and dP/dt_min_ post-arrest, indicating normal LV relaxation. Taken together, this can imply that LV dysfunction after ECMO-CPR is a failure of myocardial contractile function, not predominantly related to LV relaxation ability. Increased HR and dP/dt_max_ with reduced SV, and preserved diastolic function with maintained dP/dt_min_, tau, and end-diastolic pressure-volume relationship have also been demonstrated after hypoxic cardiac arrest in pigs [27]. Early post-arrest LV dysfunction in pre-arrest healthy hearts thus seems similar whether cardiac arrest is induced electrically or by hypoxia.

The reduced SV post-arrest was compensated for by an increased HR, maintaining CO and D_O2_. However, the substantial drop in mixed venous oxygen saturation indicates that D_O2_ was insufficient for the increased post-arrest metabolic needs. The increased V_O2_ may be due to recovery from oxygen debt and activation of other oxygen consuming processes such as systemic inflammation, induced by ischemia and reperfusion [39].

### Limitations

Our experimental model was different from a clinical ECMO-CPR scenario. In our study, healthy pigs were included, and cardiac arrest was introduced electrically. The duration of no-flow-exceeding usual clinical limits was intended to achieve a combination of survival and a certainly measurable post-arrest cardiac dysfunction. Cardiovascular function was in focus, and no attention was paid to neurological or other organ outcome. Pre-arrest heparinisation is a common experimental approach that also differs from the later on (or omitted) clinical heparin administration. Sham-operated animals were decided not included as the experiments were completed without complications, haemodynamic stability was adequate (Fig. 1), and veno-arterial ECMO only does not seem to affect intrinsic function of the porcine heart [40]. The reversibility of post-arrest myocardial LV dysfunction (stunning) was not addressed as it may take several days for the heart to fully recover. Some myocardial necrosis cannot be excluded, encouraging future investigations of myocardial damage related to ECMO-CPR in this model.

## Conclusions

An ECMO-CPR strategy of 150-min veno-arterial ECMO aiming at high blood flow rate and pharmacologically sustained aortic blood pressure and pulse pressure allowed successful resuscitation of long-lasting cardiac arrest with adequate post-arrest haemodynamic stability in pigs. MRI imaging of the in vivo beating heart demonstrated a severe early post-arrest LV dysfunction. Systolic LV function variables were substantially reduced whereas diastolic function was preserved. Cardiac MRI will be a valuable tool for future research on post-arrest cardiac dysfunction including assessment of cardiac outcome after ECMO-CPR.

## Additional files

Additional file 1:
**Table S1.** (DOCX 14 kb) Additional file 2:
**Cardiac MRI.** (DOCX 13 kb) Additional file 3:
**Table S2.** (DOCX 29 kb) Additional file 4:
**Figure S1.** (TIFF 17954 kb) Additional file 5:
**Figure S2.** (JPEG 3675 kb) Additional file 6:
**Figure S3.** (JPEG 6822 kb) Additional file 7:
**Figure S4.** (TIFF 14029 kb) Additional file 8:
**Figure S5.** (TIFF 5090 kb)

## Abbreviations

HR: heart rate
CO_phase_ and SV_phase_: cardiac output and stroke volume by cardiac MRI phase-contrast technique
EDV: end-diastolic volume
ESV: end-systolic volume
Wall thickening _septum_ and Wall thickening _lat_: mid left ventricular radial wall thickening in septum and lateral wall
MAPSE_antsept_ and MAPSE_postlat_: mitral annular plane systolic excursion measured anteroseptally and posterolaterally
HR_PA,_ CO_PA_ and SV_PA_: pulmonary artery catheter assessments of heart rate, cardiac output and stroke volume
MAP: mean aortic pressure
CVP: central venous pressure
Wedge: pulmonary arterial wedge pressure
SVR: systemic vascular resistance
LVP_max_: systolic left ventricular pressure maximum
EDP: end diastolic pressure
dP/dt_max_ and dP/dt_min_: maximum and minimum LVP time derivates
tau: tsovolumetric relaxation constant
Ea: arterial elastance
DD: diastolic duration
SD: systolic duration
IVC: isovolumetric contraction time
S_V_O_2_: mixed venous oxygen saturation
D_O2_: oxygen delivery
V_O2_: oxygen consumption
