# Supplementary material for: Successful ECMO-cardiopulmonary resuscitation with the associated post-arrest cardiac dysfunction as demonstrated by MRI
Source: Intensive Care Med Exp. 2015 Sep 3;3:25. doi: 10.1186/s40635-015-0061-2 (PMC4558998; doi:10.1186/s40635-015-0061-2)
Supplement: Additional file 1: — Table S1. (DOCX 14 kb) [file 40635_2015_61_MOESM1_ESM.docx]

| **Table S1: Detailed information on the experimental animals according to ARRIVE^*^ guidelines** | |
| --- | --- |
|  |  |
|  |  |
| **Item** | **Explanation** |
|  |  |
| Species and strain | Sus scrofa domestica, crossbreeding of Norwegian Landrace/Yorkshire sow with Norwegian Landrace/Duroc boar |
|  |  |
| Age | 4 to 6 months |
|  |  |
| Origin | Open-air farm (Østfold, Norway) |
|  |  |
| Health status | Healthy and untroubled appearance, clean and tended with no signs of infections (no diarrhoea, no infected scratching marks) |
|  |  |
| Housing | Pig boxes (2.4 m^2^) with raised floors in a 40 m^2^ room |
|  |  |
| Bedding | Aspen bedding (B&K Universal Ltd, Hull, UK) |
|  |  |
| Housing atmosphere | Carefully regulated room temperature (20**°**C) and humidity (55.7%) with 12 hours light and dark cycles. |
|  |  |
| Feeding | Unlimited access to commercial pig feed, fasted overnight with free access to tap water |
|  |  |
| Acclimatization period | One day and night |
|  |  |
| Transport to operating theatre | Premedicated in the pig box , covered by warm blanket, weighted and and weeled on trolley (approx 5 min) to the operating theatre |
|  |  |
| Operating theatre atmosphere | Sterile and stable environment with regulated temperature (20°C) and humidity (50-60 %). |

***** Animals in Research: Reporting In Vivo Experiments:

Kilkenny C, Browne WJ, Cuthill IC, Emerson M, Altman DG. Improving bioscience research reporting: the ARRIVE guidelines for reporting animal research. PLoS biology 2010;8:e1000412.
