# Supplementary material for: Successful ECMO-cardiopulmonary resuscitation with the associated post-arrest cardiac dysfunction as demonstrated by MRI
Source: Intensive Care Med Exp. 2015 Sep 3;3:25. doi: 10.1186/s40635-015-0061-2 (PMC4558998; doi:10.1186/s40635-015-0061-2)
Supplement: Additional file 2: — Cardiac MRI. (DOCX 13 kb) [file 40635_2015_61_MOESM2_ESM.docx]

**Supplement 1. Cardiac MRI**

The MRI sequences used in the experiments are presented in TableS1. Examples of cardiac MRI images from the experiments are shown in supplementary figures (Fig. S1, Fig. S2, Fig. S3, Fig. S4 and Fig. S5) to illustrate the principles of cardiac MRI measurements.

**Volumetric MRI measurements**

The LV volumes were assessed by parallel short-axis slices covering the full volume of the LV. The endocardium and epicardium of all short axis slices were manually delineated in ED and in ES as illustrated in Fig. S1. ED and ES were manually selected as the two time-frames (out of a total of 30 time-fames/beat) with the smallest and largest volumes, respectively. The most basal slice in the atrioventricular plane displayed myocardium in more than 50% of the circumference in ED. Due to the LV longitudinal contraction the mitral annular plane descends towards the apex in systole. This is known as the mitral annular plane systolic excursion, MAPSE. Because of this motion a portion of the basal cardiac MRI short axis slice(s) from the ED time-frame was not included in the ES time-frame. This is illustrated in Fig. S1 showing two basal slices excluded in total (grayed out) in the specific porcine heart used in the example. Papillary muscles and the LV outlet tract to the level of the aortic cusps were included as part of the LV volume. Stroke volume (SV) was calculated from volumetric measurements as EDV-ESV. Cardiac output (CO) was calculated volumetrically as SV ∙ heart rate. The LV ejection fraction (EF) was calculated as SV/EDV. Fig. S2 illustrates how a stack of short axis slices constitute LV volume.

**MAPSE**

MAPSE was measured manually on long axis slices (Fig. S3) and calculated as a mean of anteroseptal (MAPSE_antsep_) and posterolateral (MAPSE_postlat_) mitral annular plane excursion.

**Wall thickening**

As illustrated in Fig. S1, wall thickness (W) was measured manually in septum and lateral wall in mid LV slices perpendicular to the wall surface in ED and ES. Wall *thickening* was calculated as the percentual wall thickening from ED to ES, that is (Wall thickness in ES - Wall thickness in ED) / Wall thickness in ED. With reference to Fig. S1 this is in septum (W_ED_sept - W_ES_sept)/W_ED_sept and in lateral wall (W_ED_lat - W_ES_lat)/W_ED_lat.

**Strain**

Strain (peak global systolic LV circumferential Lagrangian strain) was assessed by harmonic phase analysis of magnetically gridded (tagged) short axis images, 20 frames/beat. This is the most widely used tool for measuring regional myocardial mechanics by MRI. The resulting peak strains in a single common time-frame from basal, mid ventricular and apical segments were averaged to define peak *global* systolic strain. Fig.S4 shows an example of tagged short axis images, with circumferential length in ED (L_0_) and ES (L_1_). Circumferential strain is defined as (L_1_-L_0_)/L_0_. In Fig.S4 the three cardiac segments are shown with their related strain curves in all 20 time-frames.

**Phase contrast imaging**

CO was also assessed by cardiac MRI flow measurements in mid ascending aorta by phase-contrast imaging. The principle of measuring CO by aortic flow may resemble the LV outlet tract velocity-time-integral by Doppler echocardiography, although the area and the mathematics of velocity measurements are very different. In principle, if volume = length ∙ area, and length/time = velocity, then flow (CO) = volume/time = velocity ∙ area. When the area of the velocity measurement is known, flow (CO) is thus provided by the velocity. Volume over time will be provided by the area under a flow-time curve. SV is then given by the area under the flow-time curve during a single systole, and CO calculated as SV ∙ heart rate.

During phase-contrast imaging, for every time-frame the velocity of blood is encoded into a separate MRI signal phase image. In every signal phase image (Fig. S5) each pixel displays a near-instantaneous blood velocity as a graded brightness. The software allows measuring and summation of pixel brightness in every signal phase image in all time-frames. With this technique blood velocity in mid aorta was calculated throughout the cardiac cycle. As the area of blood velocity encoding (mid aortic through-plane area) was also measured and manually corrected in each time-frame (40 time-frames/beat), blood flow could be plotted against time in Fig. S5. The LV stroke volume was calculated by integration of the flow-time curve from ED to ES as illustrated by the shaded area in Fig. S5.

**Legends to supplementary tables and figures**

**Legend Table S1:** **Cardiac MRI sequences**

Cardiac MRI sequences used in the experiments.

**Figure S1-S5. Principles of cardiac MRI measurements.**

**Figure S1**. Cardiac short axis slices are shown in end-diastole (ED) and end-systole (ES). Two slices in ES (grayed) were excluded from the ES volume in this example (see text). Mid LV short axis slices from ED and ES (short axis: white frames, long axis: white lines) were used to measure wall thickness (W) in septum (W_ED_sept and W_ES_sept) and lateral wall (W_ED_lat and W_ES_lat). Wall *thickening* was calculated as (W_ES_-W_ED_)/W_ED_

**Figure S2**. Left ventricular chamber volume illustrated as a stack of short axis slices.

**Figure S3**. Long axis slices in ED and ES were used to measure the mitral annular plane systolic excursion (MAPSE) toward apex in parallel with the ventricular wall. MAPSE was averaged from anteroseptal and posterolateral measurements.

**Figure S4**. Strain = (L_1_-L_0_)/L_0_ was assessed by analysis of tagged MRI images. Peak systolic strains in a single common time-frame from three cardiac segments were averaged to define peak *global* systolic circumferential strain. The three cardiac segments are shown with their related strain curves in all 20 time-frames (example from a post-arrest analysis).

**Figure S5**. Stroke volume by phase-contrast technique was provided by the area under the blood flow-time curve in systole. The area of measurement in mid ascending aorta (Ao) is illustrated in interposed long axis image and signal phase image.
