# Supplementary material for: Successful ECMO-cardiopulmonary resuscitation with the associated post-arrest cardiac dysfunction as demonstrated by MRI
Source: Intensive Care Med Exp. 2015 Sep 3;3:25. doi: 10.1186/s40635-015-0061-2 (PMC4558998; doi:10.1186/s40635-015-0061-2)
Supplement: Additional file 3: — Table S2. (DOCX 29 kb) [file 40635_2015_61_MOESM3_ESM.docx]

| **Table S2. Cardiac MRI sequences** | |
| --- | --- |
|  |  |
| **Cine** |  |
| Generic name | Spoiled gradient echo pulse |
| Images/beat | 30 |
| Spatial resolution | 1.25 x 1.25 mm |
| Slice thickness | 6 mm |
| Gap between slices | Zero |
| Short axis slices | 13-14 |
| Long axis slices | 3 |
| Repetition time (TR) | 4.2 - 4.4 msec |
| Echo time (TE) | 1.95 - 2.2 msec |
| Flip angle | 15 ° |
|  |  |
| **Tagging** |  |
| Generic name | Complementary Spatial Modulation of Magnetization (C-SPAMM) |
| Grid spacing | 8 mm |
| Images/beat | 20 |
| Repetition time (TR) | 14 msec |
| Echo time (TE) | 5 msec |
| Flip angle | 13 ° |
|  |  |
| **Phase contrast** |  |
| Generic name | Steady-state free precession (SSFP) |
| Images/beat | 40 |
| Repetition time (TR) | 5 msec |
| Echo time (TE) | 3 msec |
| Flip angle | 10 ° |
