# Supplementary figures and images for: Successful ECMO-cardiopulmonary resuscitation with the associated post-arrest cardiac dysfunction as demonstrated by MRI
Source: Intensive Care Med Exp. 2015 Sep 3;3:25. doi: 10.1186/s40635-015-0061-2 (PMC4558998; doi:10.1186/s40635-015-0061-2)

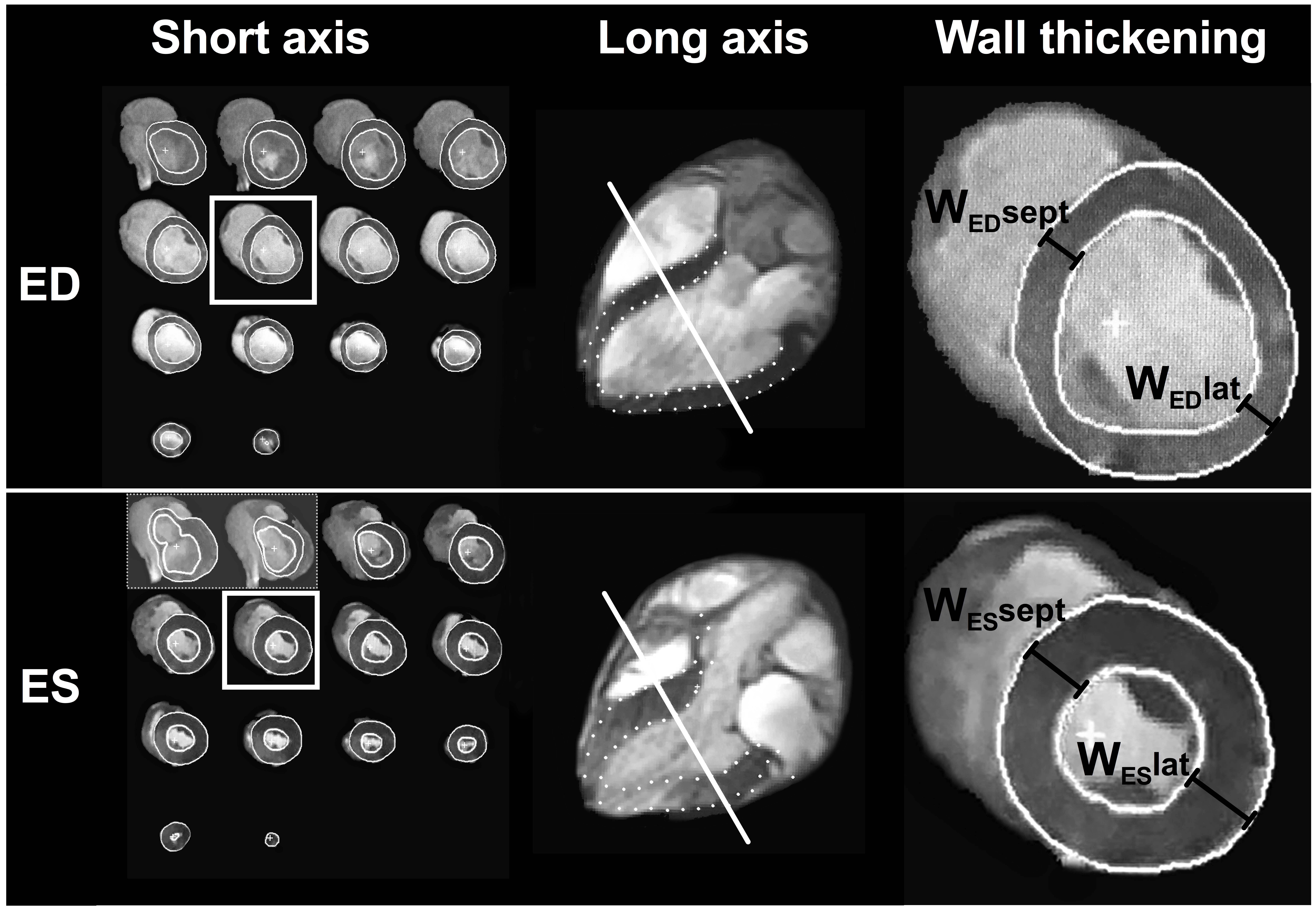

Supplement: Additional file 4: — Figure S1. (TIFF 17954 kb) [file 40635_2015_61_MOESM4_ESM.tiff]

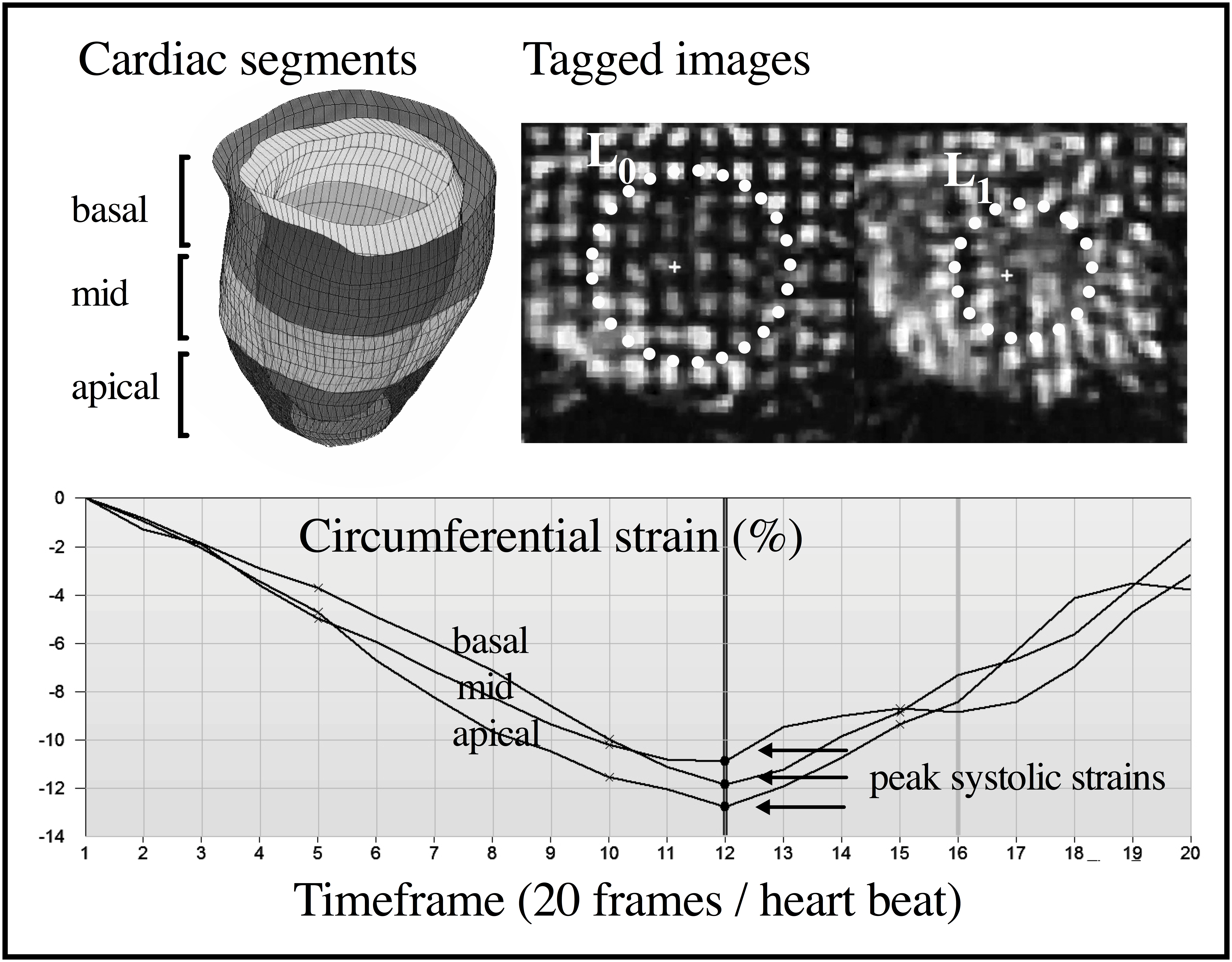

Supplement: Additional file 7: — Figure S4. (TIFF 14029 kb) [file 40635_2015_61_MOESM7_ESM.tiff]
